# MiR-27a Targets sFRP1 in hFOB Cells to Regulate Proliferation, Apoptosis and Differentiation

**DOI:** 10.1371/journal.pone.0091354

**Published:** 2014-03-13

**Authors:** Donggeng Guo, Qiuxia Li, Qing Lv, Qiujing Wei, Shuangyan Cao, Jieruo Gu

**Affiliations:** Department of Rheumatology, Third Affiliated Hospital of Sun Yat-sen University, Guangzhou, Guangdong, China; Penn State University, United States of America

## Abstract

MicroRNAs (miRNAs) play a key role in the regulation of almost all the physiological and pathological processes, including bone metabolism. Recent studies have suggested that miR-27 might play a key role in osteoblast differentiation and bone formation. Increasing evidence indicates that the canonical Wnt signaling pathway contributes to different stages of bone formation. In this study, we identify miR-27a can promote osteoblast differentiation by repressing a new target, secreted frizzled-related proteins 1 (sFRP1) expression at the transcriptional level. Here, 21 candidate targets of miR-27a involved in canonical Wnt/β-catenin signaling were predicted, and a significant decrease in sFRP1 luciferase activity was observed both in 293T and MG63 cells co-transfected with the matched luciferase reporter constructs and miR-27a mimic. Furthermore, the presence of exogenous miR-27a significantly decreased sFRP1 mRNA and protein expression in hFOB1.19 cells during both proliferation and osteogenic differentiation. The over-expression of miR-27a or knockdown sFRP1 significantly increased the percentage of apoptotic hFOBs, the percentage of cells in the G2-M phase of the cell cycle and the expression of key osteoblastic markers, including ALP, SPP1, RUNX2 and ALP activity. Over-expression of miR-27a or knockdown of endogenous sFRP1 led to an accumulation of β-catenin in hFOBs. In the present study, we demonstrate that miR-27a induced gene silencing effect is a vital mechanism contributing to bone metabolism in hFOB cells in vitro, which is partly affected by the post-transcriptional regulation of sFRP1, during osteoblast proliferation, apoptosis and differentiation.

## Introduction

Increasing evidence indicates that Wnt signaling plays a critical role in the development and maintenance of many organs and tissues, including bone [1], [2]. Wnt signaling contributes to different stages of bone formation, and the effects of Wnt signaling appear complex [3]. Thus, the regulation of bone metabolism via the canonical Wnt signaling pathway has recently become a focus of research [4], [5]. In the canonical pathway, Wnts binding to frizzled and LRP5/6 co-receptors activate canonical signaling pathways [2], [6]. Secreted frizzled-related proteins (sFRPs) contain a domain similar to frizzled proteins, which binds directly to Wnts as endogenous Wnt antagonists [7], [8]. However, the recent findings demonstrate that sFRPs can either promote or suppress Wnt/beta-catenin signaling. The promotion or suppression depends on cellular context, concentration and the expression pattern of frizzled receptors [9].

MicroRNAs (miRNAs) are short (17∼25 nucleotides) non-coding RNAs that regulate gene expression at the post- or transcriptional level [10]. Although the biological functions of most miRNAs are not yet fully understood, they may play a key role in the regulation of almost all the physiological and pathological processes [11], including osteoblastic proliferation, differentiation and death [12], [13], [14], [15]. Growing evidence supports a pivotal role for miR-27a in the multiple processes, including cancer development [16], osteogenesis [17], [15], [18], adipogenesis [19], cell proliferation [20], [21], apoptosis [22] and differentiation [23], [24]. In this study, we found that the death proportion of osteoblasts increased when the osteoblasts over-expressed miR-27a, which suggests miR-27a might be highly correlated with bone metabolism. Recently, results from several studies have supported this hypothesis [25], [26]. MiR-27a regulates the osteoblast differentiation program by siRNA specifically targeting the 3′UTR of stabilin 2 (SATB2), a powerful pro-osteoblastic transcription factor [17], [18], or significantly inhibiting adenomatous polyposis coli gene which has direct impaction on β-catenin signaling [15]. Our bioinformatics studies have predicted sFRP1 as the preferred target gene of miR-27a, but the role of miR-27a/sFRP1 in osteoblasts still remains unclear. Therefore, the current study addressed the relationship between miR-27a/sFRP1 and bone metabolism in the human fetal osteoblastic 1.19 cell line (hFOB).

## Methods

### Cell Culture

Human osteosarcoma (MG63) cells, human embryonic kidney (HEK293T) cells were purchased from the Institute of Cell Bank/Institutes for Biological Sciences (Shanghai, China, http://www.cellbank.org.cn). MG63 cells and HEK293T cells were cultured in Dulbecco’s Modified Eagle Medium (DMEM) supplemented with 10% fetal bovine serum in a humidified incubator with 95% air and 5% CO_2_ at 37°C.

The conditionally immortalized human fetal osteoblastic cell line hFOB1.19 (hFOBs) was purchased from the Institute of Cell Bank/Institutes for Biological Sciences (Shanghai, China, http://www.cellbank.org.cn). The cells were cultured according to ATCC protocol and references described as before [27], [28], [29], [30]. For in vitro proliferation, hFOB1.19 cells were maintained in the non-differentiation medium consisting of 1∶ 1 DMEM/Ham’s F-12 medium without phenol red supplemented with 10% fetal bovine serum, and 0.3 g/L G418 and were cultured in a humidified incubator with 95% air and 5% CO_2_ at 33.4°C (all from Gibco Life Technologies, NY, USA). For *in vitro* osteogenic differentiation, hFOB1.19 cells were maintained at 39.4°C or 37°C in the osteogenic medium (non-differentiation medium supplemented with β-glycerol phosphate (5×10^−3^ mol/L), ascorbic acid (0.1 g/L), menadione (10^−8 ^mol/L) and 1, 25(OH)_2_D_3_ (10^−7 ^mol/L)) (all from Sigma-Aldrich) [27], [28], [29], [30].

### Oligonucleotide Transient Transfection and Total RNA Isolation

X-tremeGENE siRNA transfection reagent (Roche Diagnostics, Shanghai, China) was used for miRNA transfection and RNA interference according to the manufacturer’s instructions (http://www.roche.com.cn). The volume and mass were based on the X-tremeGENE siRNA transfection reagent : oligonucleotides ratio of 5∶ 1. MiR-27a mimic, inhibitor and matched negative control (NC), as well as FAM-siRNAs, were synthesized by GenePharma, Shanghai, China. As previously described(http://www.roche-applied-science.com), X-tremeGENE siRNA transfection reagent was used to transfect miR-27a mimic (50 nM) or inhibitor (100 nM) and matched NC to up- or down-regulate endogenous miR-27a expression in 293T cells, MG63 cells and hFOBs. RNA interference: Four siRNA sFRP1s, which had not been validated, were also synthesized chemically by Shanghai GenePharma Co., Ltd, China. siRNA specific for sFRP1 (100 nM) and siRNA control (100 nM) were transiently transfected into hFOB1.19 cells to knock down endogenous sFRP1. The cells were lysed by adding 1 ml Trizol reagent (all from Invitrigen) for cultured cells in 10 cm^2^ dishes and passing the cell lysate several times through a pipette.

### Reverse Transcription-polymerase Chain Reaction (RT-PCR) and SYBR Green Relative Quantitative Real-time PCR

The total RNA was reverse-transcribed to cDNA using a stem-loop RT primer specific to miR-27a and real-time PCR was performed with a special primer supplied by Shanghai GenePharma Co. Ltd., by a Light Cycler PRISM 7500 (Applied Biosystems). Each sample was analyzed in triplicate. The 2^−ΔΔCt^ method was used to determine the relative gene expression [31].

### Bioinformatics

Four bioinformatic algorithms, namely TargetScan (http://www.TargetScan.org), miRBase (http://microrna.sanger.ac.uk), PicTar (http://pictar.mdc-berlin.de) and Miranda (http://www.microrna.org/microrna/home.do) were combined to predict miR-27a target genes and binding sites. To identify functional clustering annotations, the lists of candidate target genes were input into the following web-based tools: Panther (http://www.pantherdb.org), GeneCodis (http://genecodis.dacya.ucm.es/an alysis ) and Ingenuity (http://www.ingenuity.com).

### Vector Cloning and Dual-luciferase Activity Assay

The pmirGLO dual-luciferase miRNA target vector (Promega) was digested with *Sal* I and *Sac* I (TaKaRa) [32]. First, we designed and synthesized 23 pairs of short oligonucleotides containing binding sites for miR-27a in the 3′UTR of 21 candidate target genes. The multiple cloning sites of the vectors (named “pmirGLO-3′UTR (short)”) were introduced by the generated fragments annealed from short oligonucleotides. Next, 6 pairs of fragments (192∼858 bp) with miR-27a binding sites in the 3′UTR of 6 candidate target genes were amplified by PCR and inserted into the multiple cloning sites of pmirGLOs (named “pmirGLO-wt-3′UTR”) as described above to construct the wild-type recombinant plasmid. Last, to further verify the binding sites, sFRP1 fragment containing all sequences of the wt-sFRP1 3′UTR fragment except one mutant gene was inserted into the multiple cloning site of pmirGLO (named “pmirGLO-mut-3′UTR”). Luciferase activity was performed 36 h after transfection with the dual-luciferase reporter assay system (Promega), as previously described.

### Intracellular Alkaline Phosphatase (ALP) Activity

ALP activity was determined by the ALP assay kit (Abnova) according to the manufacturer’s instructions. On the test day, the cells were disrupted and corrected by total protein content with the BCA assay kit (KeyGEN,). The absorbance was read at 405 nm in a microplate reader (MK3, Thermo).

### Staining of ALP and Mineralized Matrix

ALP staining was performed by the BCIP/NBT alkaline phosphatase color development kit (Beyotime). Calcium deposits in the extracellular matrix (ECM) were detected by the argentaffin Von Kossa staining kit (GenMed) according to the manufacturer’s instructions. Images were captured at each time point with a light microscope.

### Cell Proliferation and Apoptosis Assay

The hFOB1.19 cell proliferation was measured by the CCK-8 assay kit (Dojindo) according to the manufacturer’s instructions and the absorbance was measured at 450 nm with a microplate reader (MK3, Thermo). Apoptosis analysis was measured by Annexin-V-FLUOS staining kit (Roche) and on a flow cytometer using 488 nm excitation and a 515 nm band pass filter for fluoresce in detection and a filter (>600 nm) for propidium iodide (PI) detection.

### Western Blotting

The total proteins were extracted with lysis buffer (keyGEN) for 20 min on ice. After being blocked for 1 h at room temperature, the polyvinylidene fluoride (PVDF) membranes were incubated with primary antibodies (against sFRP1, 1∶600, Abcam, USA; against β-catenin, 1∶500, Santa Cruz) overnight at 4°C and then incubated with the matched secondary antibody β-actin (goat anti-rabbit IgG, 1∶5000; goat anti-mouse IgG, 1∶5000, all from Sigma-Aldrich). The experiment was repeated at least three times.

### Immunocytofluorescence

The cells were fixed with 4% paraformaldehyde (PFA) for 10 min at room temperature and then permeabilized in 1% Triton X-100 in phosphate-buffered saline (PBS) for 15 min. After being blocked in 3% bovine serum albumin (BSA) for 30 min, the cells were incubated overnight at 4°C with the primary antibody against sFRP1 (1∶250) or β-catenin (1∶200) described as above. The secondary antibodies, dylight 488-conjugated affinipure goat anti-rabbit IgG for sFRP1 and dyligh 594-conjugated affinipure goat anti-mouse IgG for β-catenin (all from Zhongshan Gold Bridge), were added at a 1∶100 dilution and incubated for 1 h at 37°C away from light. The cells were stained for 5 min with the hoechst stain (Beyotime).

### Statistical Analysis

The comparison between experimental groups was analyzed by a Student’s t-test using SPSS 17.0 statistical software. All values were expressed in terms of mean ± SD and significant *p* values were less than 0.05 by two-tailed test.

## Results

### Relative Expression of Endogenous miR-27a in the Proliferative and Osteogenic hFOBs in vitro

**Figure 1:**
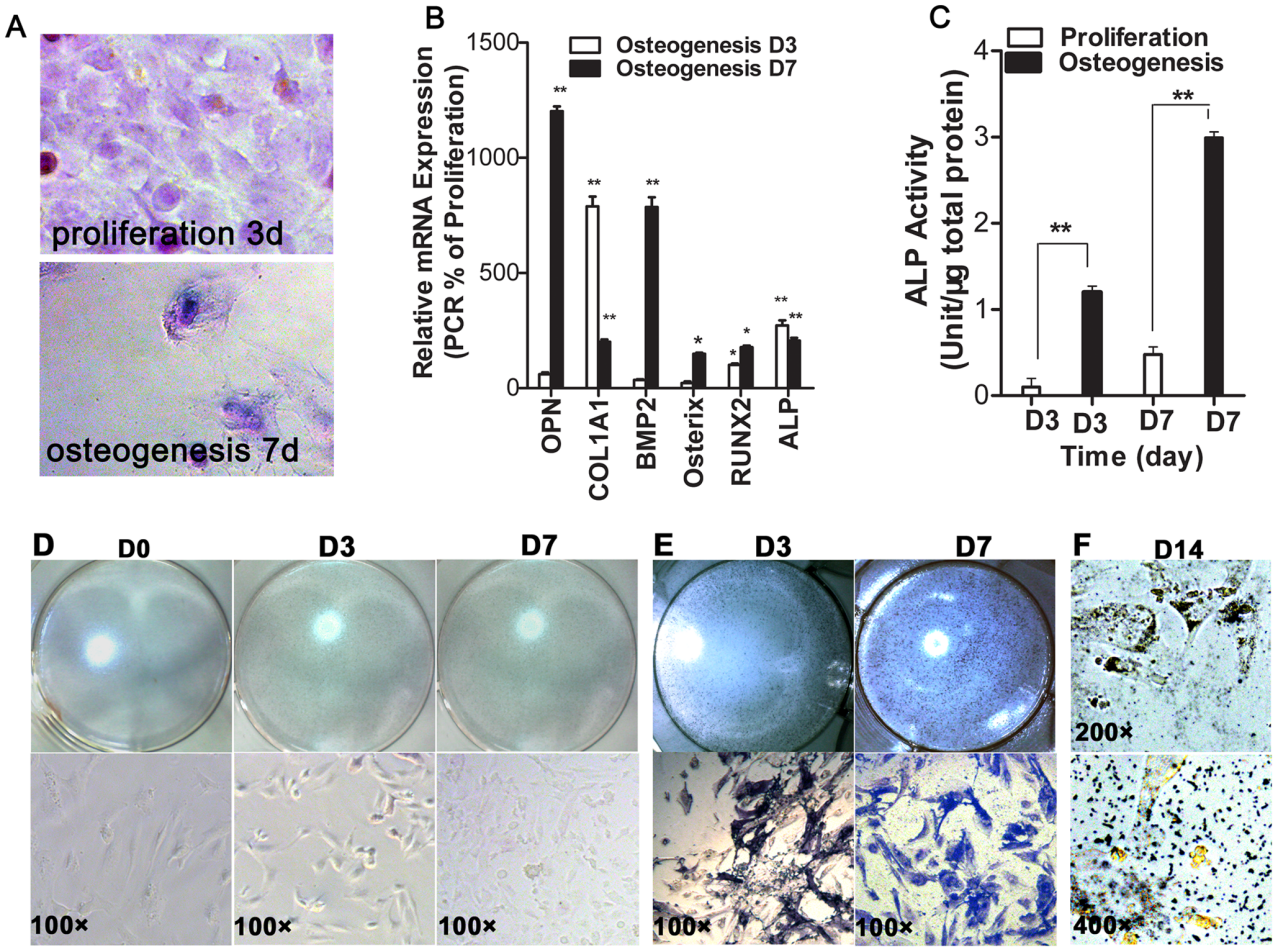
The evaluation of the optimal proliferative and osteogenic cultural condition for hFOBs *in vitro*. (**A**), After 3 days in the proliferative culture, hFOBs became small, uniform, cuboidal or spindle-shaped and closely packed with scant cytoplasm, as observed under the inverted light microscope. After 7 days in the osteogenic culture, hFOBs became larger and uneven in size, with abundant cytoplasm and shortened processes. Compared with hFOBs in the non-differentiation medium at 33.4°C for 3 or 7 days, hFOBs cultured in the osteogenic medium at 39.4°C exhibited obvious increases in the mRNA expression of bone-related genes (including ALP, COL1A1, OPN, Osterix, RUNX2 and BMP2) (**B**), as well as increased ALP activity (**C**), ALP staining (**D** and **E**) and calcium deposition staining (**F**). N = 3; mean ± SD; *p<0.05, **p<0.01.

**Figure 2:**
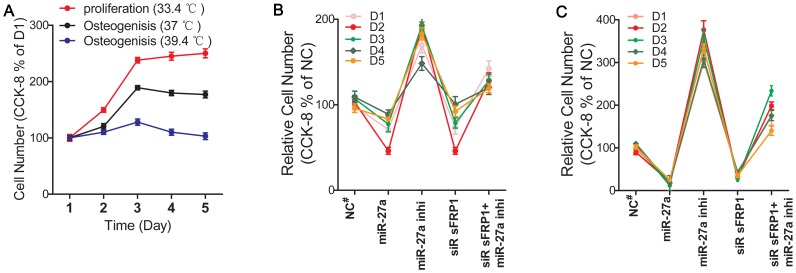
HFOB proliferation assay. The proliferation was assessed by the CCK-8 active cell number kit. (**A**), hFOBs at the same passage were seeded into 96-well plates at a density of 7, 000 cells/well and cultured for 1 to 5 days in the non-differentiation medium at 33.4°C (red curve) or in the osteogenic medium at 37°C (black curve) or 39.4°C (blue curve). At 33.4°C, the daily cell counts indicated exponential cell growth between days 2 and 3, and the doubling time was approximately 60 h. (**B** and **C**), the hFOBs were transfected with the miR-27a mimic, miR-27a inhibitor or siRNA sFRP1 or were co-transfected. After 8 h, the cells either remained in the non-differentiation medium at 33.4°C (**B**) or were transferred to the osteogenic medium at 39.4°C (**C**). The proliferative capability of the hFOB cells was expressed as the percentage of surviving cells in comparison to the matched control. N = 9 for each group. The values represent the mean ± SD. NC^#^ was the matched control: miR NC for miR-27a mimic or siRNA sFRP1; miR inhibitor NC for miR-27a inhibitor; siR-sFRP1+ miR-27a inhibitor NC for siR sFRP1+ miR-27a inhibitor.

**Figure 3:**
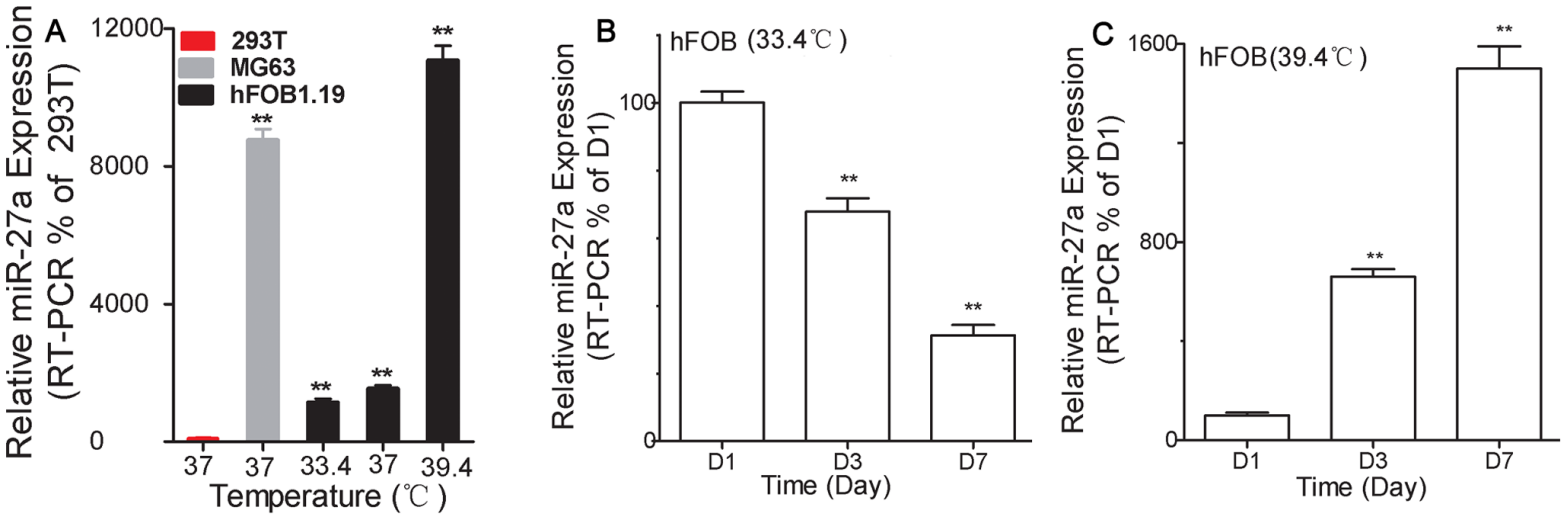
Relative expression of the endogenous miR-27a in different cell types. Relative miR-27a expression was determined after 48 h among three different cell types (**A**) and different time (**B** and **C**) under the conditions described as follows. MG63 and 293T cells were cultured in DMEM supplemented with 10% fetal bovine serum at 37°C for up to 3 days (**A**). HFOB1.19 cells were maintained in the non-differentiation medium at 33.4°C (**B**) or in the osteogenic medium at 39.4 (**C**) for up to 3 days or 7 days. All data are shown as mean ± SD. The asterisk indicates significance (t-test, **p<0.01).

Firstly, we evaluated that the ideal condition of proliferation and osteogenesis *in vitro* for hFOBs by examining cell morphology, growth curves, osteoblast markers, ALP activity, ALP staining and calcium deposition (Figure 1 and 2). The findings indicate that the ideal proliferation *in vitro* for hFOBs occurs in the non-differentiation medium at 33.4°C and the optimal osteogenesis occurs in the osteogenic medium at 39.4°C, as previously shown [27], [28], [33], [34], [35], [36]. Then, we detected relative expression of endogenous miR-27a in the proliferative and osteogenic hFOBs *in vitro* compared to 293T as negative control and MG63 cells as positive control using a stem-loop real-time PCR (Figure 3). MiR-27a expression was higher in MG63 cells and was the highest in osteogenic hFOBs compared to 293T cells. During the 3 days’ culture, 293T cells showed no time-dependent variation in the expression of miR-27a. MiR-27a expression in MG63 cells was up-regulated after 2 days of proliferation (*p*≤0.01). However, miR-27a expression in hFOBs was significantly time-dependent down-regulated (*p*≤0.01) during the 7-day proliferation and was time-dependent up-regulated during the osteogenic differentiation. The experiments suggested that the over-expression of miR-27a in hFOBs was due to osteogenesis.

### Prediction of hsa-miR-27a Targets and Binding Sites

The miR-27 family includes miR-27a and miR-27b, of which hairpin precursors are quite different, whereas their mature forms are identical except for the distinction on a single nucleotide (http://www.miRBase.org, Release 16: April 2010). There were 923, 1119, 761 and 7800 miR-27a candidate targets predicted by TargetScan, miRBase, PicTar and Miranda, respectively. The intersection of any two of these four algorithms contained 1433 candidate miR-27a target genes. Functional clustering annotations showed that Wnt/β-catenin signaling pathway was one of the putatively co-regulated biological processes (Table S1–S3). 21 targets (containing 23 binding sites) of miR-27a within the Wnt/β-catenin pathway were validated to have perfect complementarity with the miRNA seeds by TargetScan database (http://www.Target Scan.org, version 5.1∶2009) [37].

### Dual-luciferase Activity Assay for the Validation of miR-27a Direct Interactions with the 3′UTRs of Target Genes

**Figure 4:**
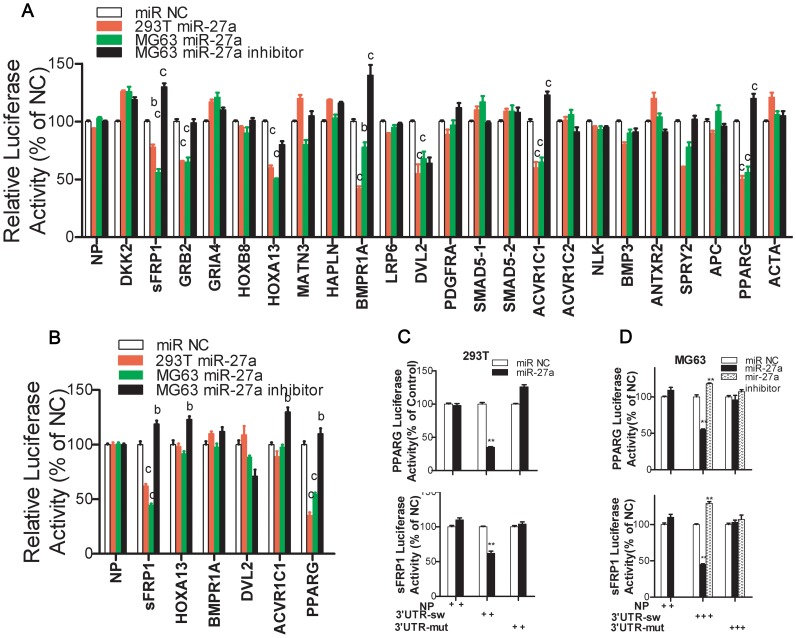
Dual-luciferase activity assay showing the direct interaction between miR-27a and sFRP1-3′UTR. (**A**), 23 luciferase reporter recombinant plasmids carrying short oligonucleotides (35 bps) which can flank the conserved miR-27a seed sequence were transfected into 293T or MG63 cells for the dual-luciferase assay to perform a primary screen for miR-27a target genes. In the presence of miR-27a, luciferase activity was significantly decreased in Luc-sFRP1, Luc-HOXA13, Luc-BMPR1A, Luc-DVL2 and Luc-ACVR1C1 3′UTR reporter constructs. (**B**), The Luc-sFRP1, Luc-HOXA13, Luc-BMPR1A, Luc-DVL2 and Luc-ACVR1C1 3′UTR reporter constructs containing longer fragments (376∼858 bps) or the entire 3′UTRs synthetized using PCR were transfected into 293T and MG63 cells again for luciferase assay to further screen miR-27a target genes. MiR-27a specifically interacts with the 3′UTR of wild-type sFRP1 mRNA but not mutant sFRP1 (**C** and **D**). All values are given relative to transfections with the appropriate negative control (NC) oligonucleotide of miR-27a. Luciferase assay for *PPARG* served as a positive group and for naked pmirGLO (NP) vector as a negative group. The data represent the average of three separate transfections performed in triplicate on different days with cells after 3–5 passages. N = 9. Mean ± SD. ^b^p or^ *^p≤0.05; ^c^p or ^**^p≤0.01**vs. NC group.

23 recombinant plasmids named pmirGLO-3′UTRs (short) containing the 23 binding sites of 21 miR-27a target genes were constructed for the dual-luciferase assay and identified following digestion with *Eco*R V and sequencing. We up-regulated miR-27a expression in 293T cells which were lacked the endogenous miR-27a. We also performed miR-27a up-regulation and knockdown in MG63 cells which had a relatively high endogenous expression of miR-27a (Figure 3). To control the variability in the transfection efficiency of the miR-27a mimic or inhibitor in 293T and MG63 cells, optimization and validation of the transfection system were performed (Figure S1). In 293T and MG63 cells co-transfected with the pmirGLO-3′UTRs (short) and miR-27a mimic, the luciferase activity, including Luc- PPARG (as positive control) [24], [38], Luc-GRB2, Luc-HOXB13, Luc-BMPR1A, Luc-DVL2, Luc-sFRP1 and Luc-ACVR1C1, was markedly lower than that of the naked pmirGLO plasmid (as negative control) (Figure 4). In contrast, the luciferase activity, including Luc-sFRP1, Luc-BMPR1A and Luc-ACVR1C1 in MG63 cells transfected with the miR-27a inhibitor was significantly higher than that of the negative control (Figure 4). The luciferase activity assay of pmirGLO-wt-3′UTRs containing longer fragments or the entire 3′UTRs showed that pmirGLO-wt-sFRP1-3′UTR exhibited significantly down-regulated luciferase activity in both 293T and MG63 cells with over-expressed miR-27a (Figure 4). Further, the luciferase activity in 293T and MG63 cells transfected with miR-27a and pmirGLO-mut-sFRP1-3′UTR was not significantly down-regulated than the negative control (Figure 4).

### MiR-27a Negatively Regulates sFRP1 Expression at the Transcriptional Level in vitro

**Figure 5:**
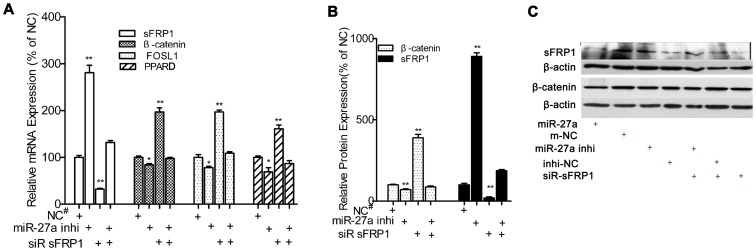
MiR-27a negatively regulates sFRP1 expression and activates the canonical Wnt/β-catenin signaling pathway. (**A**), Real-time PCR analysis of endogenous sFRP1, β-catenin, PPARD or FOSL1 mRNA level in hFOBs transfected with miR-27a mimic, inhibitor, or sFRP1 siRNA in the osteogenic medium at 39.4°C. (**B** and **C**), Western blotting analysis for the endogenous β-catenin and sFRP1 protein level, respectively, in hFOBs transfected with miR-27 mimic, inhibitor or sFRP1 siRNA. HFOBs were cultured in the non-differentiation medium at 39.4°C. N = 3; mean ± SD; *p<0.05; **p<0.01. NC^#^ was the matched control: miR NC for miR mimic or siR sFRP1; miR inhibitor NC for miR-27a inhibitor; siR-sFRP1+ miR-27a inhibitor NC for siR sFRP1+ miR-27a inhibitor. m-NC: miR mimic control. Inhi-NC: miR inhibitor control.

To determine whether the miR-27a regulates sFPR1 expression directly, we use real-time PCR to evaluate the *sFRP1* mRNA expression and use Western Blotting to detect the sFRP1 protein expression in proliferative and osteogenic hFOBs. Real-time PCR analysis showed there was a significant reduction in the *sFRP1* mRNA level in hFOBs transfected with the miR-27a mimic (32.61% in the proliferative model, *p*<0.01; 20.88% in the osteogenic model, *p*<0.01) and an increase greater than 2 folds in hFOBs transfected with the miR-27a inhibitor as compared to hFOBs transfected with miRNA control (*p*<0.01). Furthermore, miR-27a inhibitor-induced up-regulation of *sFRP1* can be partly counteracted by siRNA-induced reduction in *sFRP1* expression (Figure 5).

### MiR-27a is a Novel Regulator of Preosteoblast Proliferation

The CCK-8 cell assay (Figure 2 and Table S4–S7) demonstrated that over-expression of miR-27a or knockdown of endogenous *sFRP1* significantly inhibited the proliferation of hFOBs in both non-differentiation and osteogenic differentiation conditions from day 1 to day 5 of culture (*p*<0.01). However, transfection with miR-27a inhibitor to inhibit endogenous miR-27a led to a greater increase in hFOB proliferation compared to transfection with the miR-27a inhibitor control (*p*<0.01). Furthermore, miR-27a inhibitor-induced up-regulation of proliferation can be partly counteracted by siRNA-sFRP1-induced reduction in proliferation.

### MiR-27a Facilitating Preosteoblast Apoptosis by sFRP1 Inhibition

**Figure 6:**
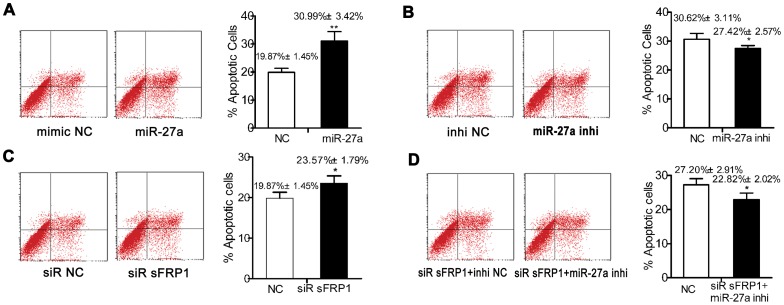
MiR-27a facilitates apoptosis by inhibiting sFRP1 in hFOB cells. HFOBs were plated in 6-well microplates (2.6×10^5^ cells/well) and incubated in the non-differentiation medium at 33.4°C for 24 h to 75% confluence. The hFOBs were then transfected with the miR-27a mimic (50 nM) (**A**), miR-27a inhibitor (100 nM) (**B**), siRNA sFRP1 (100 nM) (**C**), or matched negative control. Some cells were co-transfected in parallel with the miR-27a inhibitor (100 nM) and siRNA sFRP1 (100 nM), or matched negative control (**D**). The percentage of apoptotic hFOBs was determined by FACS analysis, as described in the materials and methods, at 48 h after transfection and incubation in the non-differentiation medium. N = 6 for each group. The values represent the mean ± SD. *p<0.05; **p<0.01.

Transfection with miR-27a mimic significantly increased the percentage of apoptotic hFOBs, whereas inhibition of endogenous miR-27a significantly decreased the percentage of apoptotic hFOBs. In addition, the knockdown of endogenous *sFRP1* resulted in a significant increase in the percentage of apoptotic hFOBs. Interestingly, miR-27a inhibitor-induced increase of apoptotic hFOBs can be partly counteracted by siRNA-sFRP1-induced decrease in apoptotic hFOBs (Figure 6).

### MiR-27a Blocks Cells at G2-M Phase

**Figure 7:**
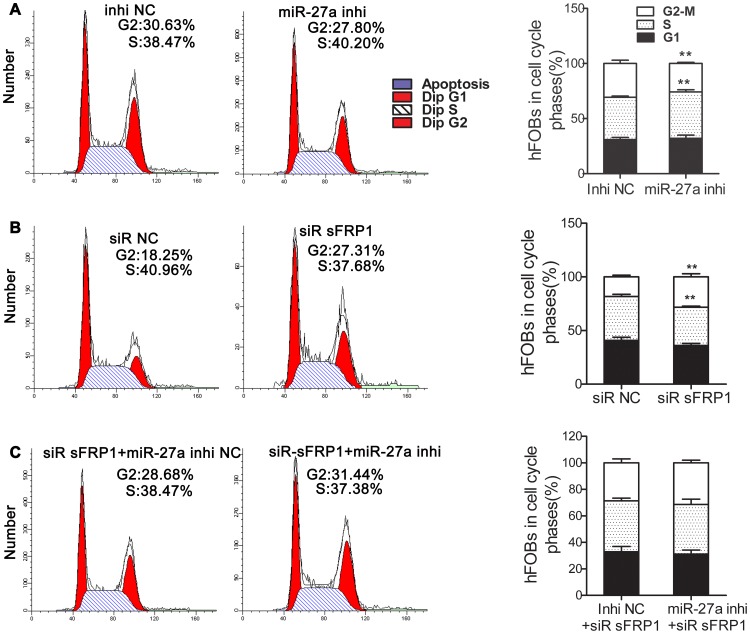
Modulation of cell cycle progression by miR-27a and sFRP1. HFOBs were plated in 6-well microplates (2.6×10^5^ cells/well) and incubated in the non-differentiation medium at 33.4°C for 24 h to 75% confluence. hFOBs were then transfected with the miR-27a inhibitor (100 nM) (**A**), siRNA sFRP1 (100 nM) (**B**) or both (**C**), or the matched control (at the same concentration). The percentage of cells in each phase of the cell cycle was determined by FACS analysis, as described in the materials and methods, at 48 h after transfection. N = 6 for each group. The values represent the mean ± SD. **p<0.01.

MiR-27a knockdown both increased the percentage of hFOBs in the S phase of the cell cycle and decreased the percentage of cells in G2-M phase. However, *sFRP1* knockdown decreased the percentage of cells in the S phase and increased the percentage of cells in G2-M phase. Moreover, the effects caused by *sFRP1* knockdown were alleviated in hFOBs transfected with the miR-27a inhibitor (Figure 7).

### MiR-27a Promotes Osteoblastic Differentiation by Directly Inhibiting sFRP1 Independent of the Culture Medium

**Figure 8:**
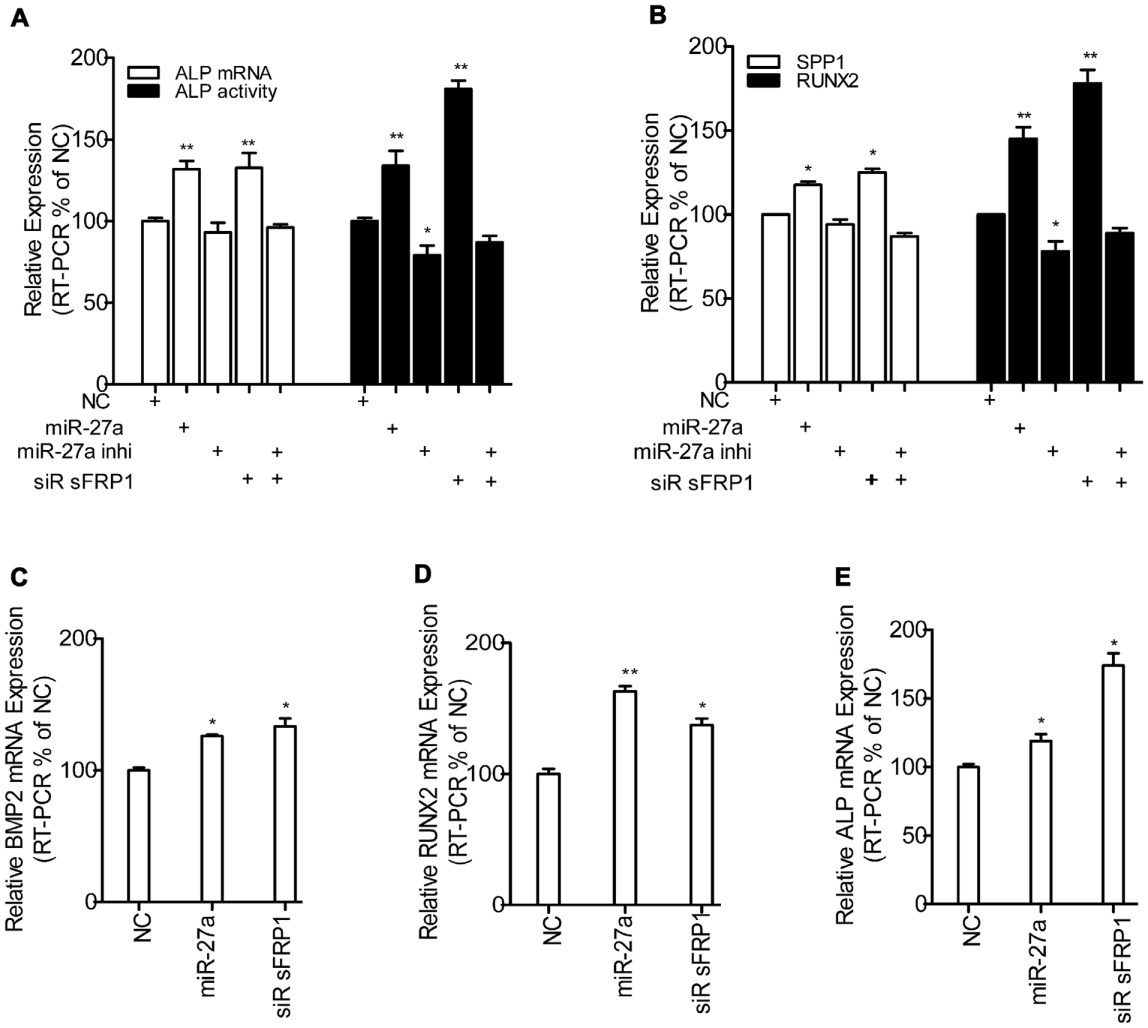
The effect of miR-27a/sFRP1 on osteoblast markers in osteogenic hFOBs. ALP (**A**), SPP1 and RUNX2 mRNA (**B**) expression was determined by real-time PCR, and ALP activity (**A**) was assessed in hFOBs cultured in the osteogenic medium at 39.4°C. BMP2 (**C**), RUNX2 (**D**) and ALP (**E**)mRNA expression in hFOBs cultured in the non-differentiation medium at 39.4°C was also evaluated by real-time PCR. N = 3; mean ± SD; *p<0.05; **p<0.01. NC was the matched negative control: miR NC for both miR-27a mimic and siR sFRP1; miR inhibitor NC for miR-27a inhibitor; siR sFRP1+ miR-27a inhibitor NC for siR sFRP1+ miR-27a inhibitor.

As shown in Figure 3, miR-27a expression was the highest in osteogenic hFOBs and was significantly down-regulated during the proliferation and up-regulated during the osteogenic differentiation of hFOBs. Furthermore, hFOB cells transfected with the miR-27a mimic or siRNA sFRP1, respectively, showed a significant increase in osteoblastic marker, both in the osteogenic medium and non-differentiation medium, compared to the matched control. In contrast, hFOBs transfected with the miR-27a inhibitor and then grown in the same conditions demonstrated significantly greater reductions in the mRNA expression of osteoblastic markers. However, the effects caused by *sFRP1* knockdown were alleviated in hFOBs transfected with the miR-27a inhibitor (Figure 8).

### MiR-27a Activates Wnt/β-catenin Signaling by Inhibiting sFRP1 *in vitro*

**Figure 9:**
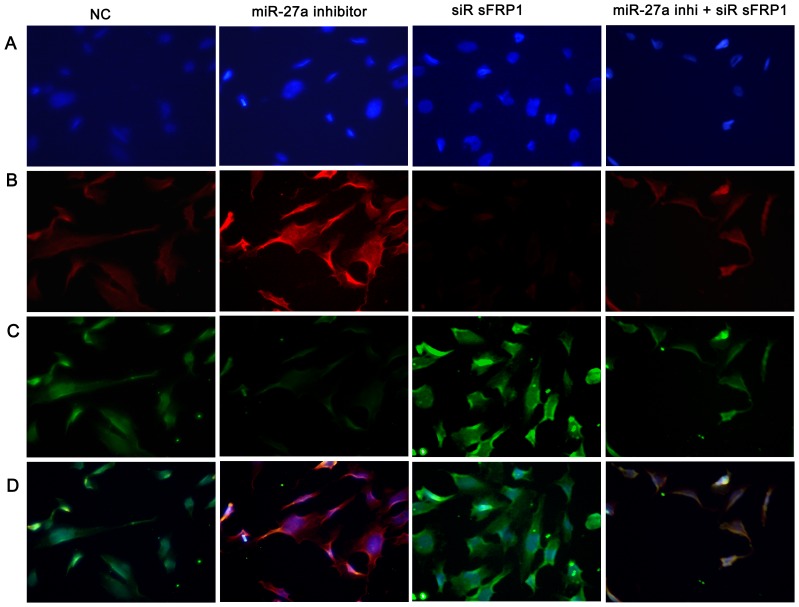
The effect of miR-27a on sFRP1 or Wnt/β-catenin signaling was observed by immunocytofluorescent staining. HFOB1.19 cells (2.7×10^4^ cells/well) were plated in 24-well plates and cultured to 90% confluence for the transient transfection. hFOBs were transfected with the miR-27a inhibitor (100 nM) or siRNA sFRP1 (100 nM) or were co-transfected with both of them and then cultured in the osteogenic medium at 39.4°C. Immunocytofluorescence analysis was performed 72 h after the transfection. Briefly, hFOBs were incubated overnight at 4°C with a primary antibody against sFRP1 (1∶250) or β-catenin (1∶200). The cells were then incubated with secondary antibodies, including goat-anti-rabbit IgG (red fluorescence, **B**) for sFRP1 and goat-anti-mouse IgG (green fluorescence, **C**) for β-catenin, both at a 1∶100 dilution in the dark. Hochest stain was used to stain the cell nuclei (blue fluorescence, **A**). Figure **D** depicts the superimposed images of nuclei, sFRP1 and β-catenin. The data are representative of three times independent experime.

Using real-time PCR (Figure 5), Western blotting (Figure 5) and immunocytofluorescent staining (Figure 9), we found that over-expression of miR-27a or knockdown of endogenous *sFRP1* alone caused an accumulation of β-catenin in osteogenic hFOBs at 39.4°C in both osteogenic and non-differentiation culture medium. However, the effects caused by *sFRP1* knockdown were alleviated in hFOBs transfected with the miR-27a inhibitor.

We next validated the influence of miR-27a/sFRP1 on terminal genes of the Wnt/β-catenin signaling pathway (Figure 5). The expression of *PPARD* mRNA in hFOBs transfected with the miR-27a mimic or siRNA *sFRP1* alone was markedly increased as compared to the negative control, whereas the co-transfection of miR-27a inhibitor and siRNA sFRP1 reversed the *PPARD* expression observed following the co-transfection with miRNA negative control and siRNA sFRP1. However, the effect of miR-27a to inhibit *sFRP1* led to decreased change in FOSL1 expression.

## Discussion

The primary culture of human mesenchymal stromal cells has been widely used in the cell proliferation and differentiation research *in vitro*. Although these are excellent model systems, their scarcity, heterogeneity, and limited lifespan restrict their usefulness [29]. Here we provide novel insights into the regulation of miR-27a in the conditionally immortalized human fetal osteoblastic cell line hFOB.1.19 (hFOB). The Primary cultures of hFOB1.19 cells were isolated from fetal tissue and transfected with a gene coding for a temperature-sensitive mutant of SV40 large T antigen (ts-SV40LTA) as well as a gene coding for neomycin (G418) resistance [28]. The hFOB has multilineage differentiation potential, including as a preosteoblast. Incubation of hFOB cells at the permissive temperature of 33.4°C resulted in activation of ts-SV40LTA and rapid cell proliferation. These undifferentiated hFOB cells possess similar cell surface markers as mesenchymal stromal cells. Furthermore, at the restrictive temperature of 39.4°C and the differentiate medium described as methods, ts-SV40LTA is inactive and osteogenic differentiation of hFOBs occurs [27], [28], [39]. These osteogenic hFOBs can synthesize and mineralize bone matrix and express many osteoblastic markers, including high ALP activity, mineralization, RUNX2 and BMP2 [28], [27].

Although others reported the negative regulation of a luciferase–APC or -STAB2 3′UTR construct by miR-27 in osteoblasts or mesenchymal stem cells [15], we identified a new miR-27a target, sFRP1, by different methods including bioinformatics, dual-luciferase activity assay and functional regulation. The mode of miR-27a regulating sFRP1 in hFOBs is the transcriptional level not the post-transcriptional level.

It is well established that the expression of genes which is important for the activation of canonical Wnt/β-catenin signaling is increased when the osteoblasts become more osteogenic differentiated with increased osteogenic markers such as ALP activity, mineralization, RUNX2 and BMP2 [40], [14], [41]. Our observations correlate with these genes expression patterns in that miR-27a is significantly time-dependent up-regulated during osteogenic differentiation, likely in part due to an increase in canonical Wnt/β-catenin signaling. We next evaluated the impact of miR-27a on β-catenin and the terminal genes of the canonical Wnt/β-catenin signaling pathway in hFOBs at 39.4°C in both osteogenic and non-differentiation culture medium in vitro. This finding showed that miR-27a positively regulated osteoblast differentiation by directly inhibiting the expression of sFRP1 and likely other targets [15], and thus modulates the Wnt/β-catenin signaling pathway.

In addition, we established that miR-27a expression is significantly time-dependent down-regulated during the cell proliferation. During our studies, significant cell death was observed in hFOB cells over-expressed with miR-27a or knockdown with sFRP1. Some investigators reported a similar experience that over-expression of miRNAs which can active the Wnt/β-catenin signaling, could impose a strong negative selection against cell growth in vitro [14], [42]. Consistent with this, our experiments showed that miR-27a arrested hFOBs at G2-M phase and facilitated hFOBs apoptosis, in part, by inhibiting the expression of sFRP1.

In conclusion, miR-27a induced gene silencing effect is a vital mechanism contributing to bone metabolism in hFOB cells in vitro, in partly by the transcriptional regulation of sFRP1, during osteoblast proliferation, apoptosis and differentiation. These data have important implications with regard to some disease with bone metabolism disorders.

## Supporting Information

Figure S1 **Verification of the effect of transient transfection.** The miR-27a mimic (50 nM) or miR-27a inhibitor (100 nM), labeled with a blue flourescent molecule (FAM-siRNA), were transfected into 293T cells and MG63 cells using the X-tremeGENE siRNA Transfection Reagent. The transfection efficiency of miR-27a into 293T and MG63 cells was 99.4% (**A**) and 97.2% (**C**), respectively, as assessed by flow cytometry. Relative miR-27a expression was also determined by stem-loop SYBR Green real-time PCR in 293T (**B**) and MG63 cells (**D**). Relative miR-27a expressions are shown as mean ± SD. The asterisk indicates significance (t-test, *p<0.05; **p<0.01).
(TIF)

Table S1 **Pathway analysis of miR-27a target genes - GeneCodis analysis (KEGG Pathways).**
(DOC)

Table S2 **Pathway analysis of miR-27a target genes - PANTHER analysis.**
(DOC)

Table S3 **Pathway analysis of miR-27a target genes - Ingenuity analysis.**
(DOC)

Table S4 **The influence of miR-27a/sFRP1 on hFOB proliferation. (non-differentiation** ***in vitro*** **).** (OD450, Mean ± SD).
(DOC)

Table S5 **The inhibitory effect of sFRP1 knockdown on hFOBs proliferation can be reversed by the knockdown of miR-27a.** (non-differentiation *in vitro*) (OD450, Mean ± SD).
(DOC)

Table S6 **The influence of miR-27a/sFRP1 on hFOB proliferation. (osteogenic differentiation** ***in vitro).*** (OD450, Mean ± SD).
(DOC)

Table S7 **The inhibitory effect of sFRP1 knockdown on hFOB proliferation can be reversed by the knockdown of miR-27a.** (osteogenic differentiation *in vitro*) (OD450, Mean ± SD).
(DOC)
