# Supplementary figures and images for: MiR-27a Targets sFRP1 in hFOB Cells to Regulate Proliferation, Apoptosis and Differentiation
Source: PLoS One. 2014 Mar 13;9(3):e91354. doi: 10.1371/journal.pone.0091354 (PMC3953332; doi:10.1371/journal.pone.0091354)

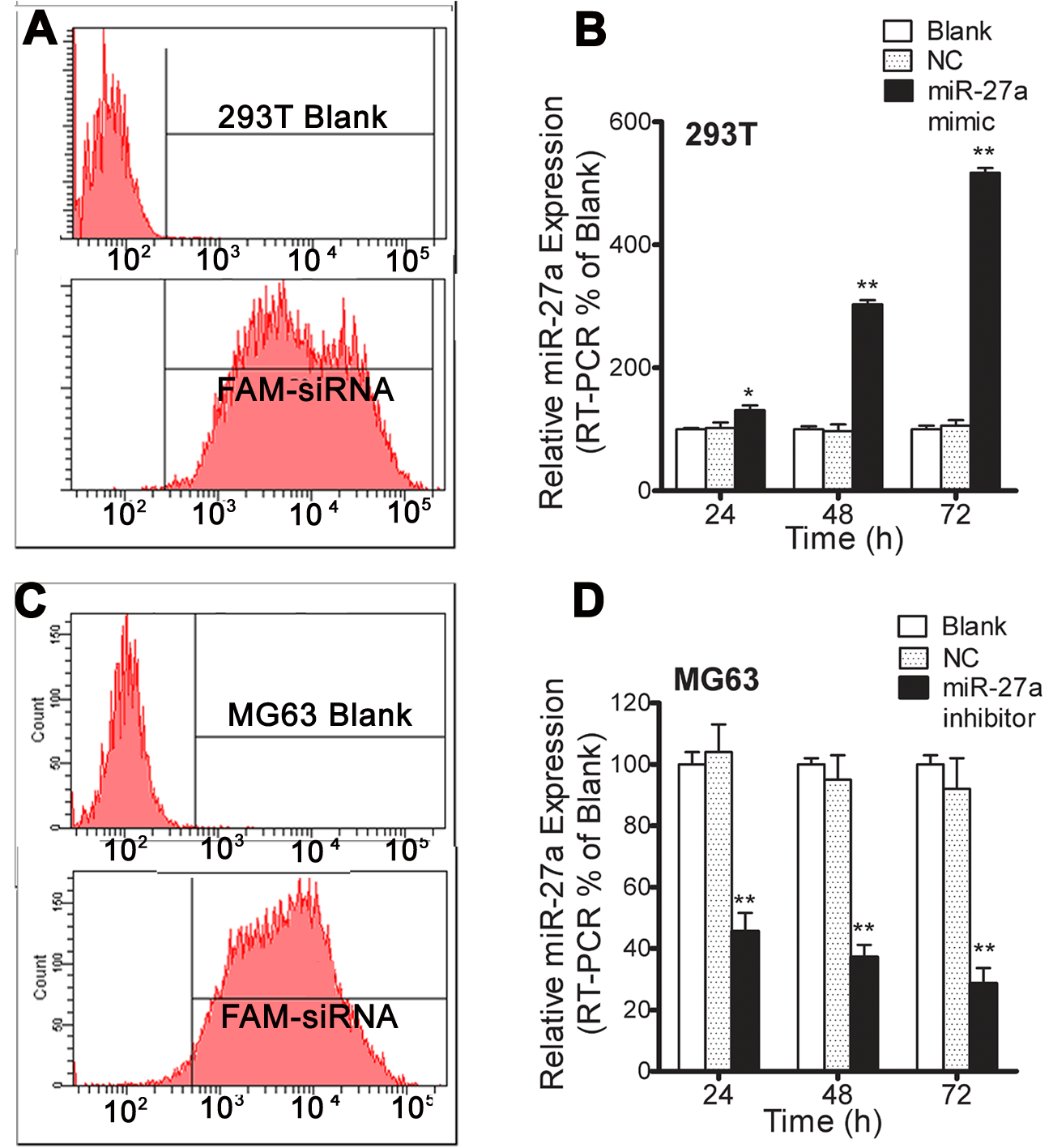

Supplement: Figure S1 — Verification of the effect of transient transfection. The miR-27a mimic (50 nM) or miR-27a inhibitor (100 nM), labeled with a blue flourescent molecule (FAM-siRNA), were transfected into 293T cells and MG63 cells using the X-tremeGENE siRNA Transfection Reagent. The transfection efficiency of miR-27a into 293T and MG63 cells was 99.4% (A) and 97.2% (C), respectively, as assessed by flow cytometry. Relative miR-27a expression was also determined by stem-loop SYBR Green real-time PCR in 293T (B) and MG63 cells (D). Relative miR-27a expressions are shown as mean ± SD. The asterisk indicates significance (t-test, *p<0.05; **p<0.01). (TIF) [file pone.0091354.s001.tif]
