# Supplementary material for: MiR-27a Targets sFRP1 in hFOB Cells to Regulate Proliferation, Apoptosis and Differentiation
Source: PLoS One. 2014 Mar 13;9(3):e91354. doi: 10.1371/journal.pone.0091354 (PMC3953332; doi:10.1371/journal.pone.0091354)
Supplement: Table S1 — Pathway analysis of miR-27a target genes - GeneCodis analysis (KEGG Pathways). (DOC) [file pone.0091354.s002.doc]

**Table S1. Pathway analysis of miR-27a target genes - GeneCodis analysis (KEGG Pathways).**

| Genes | NGR | NG | Hyp | Hyp* | Annotations |
| --- | --- | --- | --- | --- | --- |
| 47 | 265(29095) | 47(1385) | 5.53E-15 | 4.78E-13 | MAPK signaling pathway |
| 20 | 86(29095) | 20(1385) | 2.82E-09 | 9.76E-08 | TGF-β signaling pathway |
| 21 | 108(29095) | 21(1385) | 3.40E-08 | 7.36E-07 | T cell receptor signaling pathway |
| 18 | 98(29095) | 18(1385) | 7.75E-07 | 7.88E-06 | GnRH signaling pathway |
| 20 | 150(29095) | 20(1385) | 3.09E-05 | 2.54E-04 | Wnt signaling pathway |
| 20 | 155(29095) | 20(1385) | 4.96E-05 | 0.00033 | Jak/STAT signaling pathway |

This file contains the list of enriched pathways obtained using GeneCodis analysis that satisfied the criteria of a *p*-value <0.01. NGR = Number of annotated genes in the reference list (total number of genes in the reference list); NG = Number of annotated genes in the input list (total number of genes in the input list); Hyp = Hypergeometric *p* Value; Hyp* = Corrected hypergeometric *p* value.
