# Supplementary material for: MiR-27a Targets sFRP1 in hFOB Cells to Regulate Proliferation, Apoptosis and Differentiation
Source: PLoS One. 2014 Mar 13;9(3):e91354. doi: 10.1371/journal.pone.0091354 (PMC3953332; doi:10.1371/journal.pone.0091354)
Supplement: Table S2 — Pathway analysis of miR-27a target genes - PANTHER analysis. (DOC) [file pone.0091354.s003.doc]

**Table S2. Pathway analysis of miR-27a target genes - PANTHER analysis.**

| Pathways | +/- | *p* value |
| --- | --- | --- |
| Wnt signaling pathway | + | 2.75E-05 |
| FGF signaling pathway | + | 1.27E-04 |
| TGF-β signaling pathway | + | 3.03E-03 |
| p53 pathway feedback loops 2 | + | 1.37E-02 |
| JAK/STAT signaling pathway | + | 1.52E-02 |
| T cell activation | + | 3.43E-02 |
| p53 pathway by glucose deprivation | + | 3.49E-02 |

This file contains the list of enriched pathways obtained using PANTHER analysis that satisfies the criteria of p-value <0.01. +/-: overrepresentation of a category is denoted by a “+” sign and underrepresentation by a “–” sign.
