# Supplementary material for: MiR-27a Targets sFRP1 in hFOB Cells to Regulate Proliferation, Apoptosis and Differentiation
Source: PLoS One. 2014 Mar 13;9(3):e91354. doi: 10.1371/journal.pone.0091354 (PMC3953332; doi:10.1371/journal.pone.0091354)
Supplement: Table S3 — Pathway analysis of miR-27a target genes - Ingenuity analysis. (DOC) [file pone.0091354.s004.doc]

**Table S3. Pathway analysis of miR-27a target genes - Ingenuity analysis.**

| Ingenuity Canonical Pathways | -log (*p* value) |
| --- | --- |
| Wnt/β-catenin signaling pathways | 4.00 |
| p38 MAPK signaling pathways | 3.40 |
| TGF-β signaling pathways | 3.19 |
| BMP signaling pathway | 3.01 |
| NF-κB signaling pathways | 3.00 |
| T helper cell differentiation pathways | 2.24 |

This file contains the list of enriched pathways obtained using Ingenuity analysis that satisfied the criteria of a *p* <0.01, -log0.01 ≥2.00.
