# Supplementary material for: MiR-27a Targets sFRP1 in hFOB Cells to Regulate Proliferation, Apoptosis and Differentiation
Source: PLoS One. 2014 Mar 13;9(3):e91354. doi: 10.1371/journal.pone.0091354 (PMC3953332; doi:10.1371/journal.pone.0091354)
Supplement: Table S4 — The influence of miR-27a/sFRP1 on hFOB proliferation. (non-differentiation in vitro ). (OD450, Mean ± SD). (DOC) [file pone.0091354.s005.doc]

**Table S4. The influence of miR-27a/sFRP1 on hFOB proliferation. (non-differentiation *in vitro*).** (OD450, Mean ± SD)

|  | miR-27a mimic | NC^①^ | miR-27a inhibitor | NC^②^ | siR-sFRP1 |
| --- | --- | --- | --- | --- | --- |
| D1 | 0.3990±0.0080^**^ | 0.4797±0.0074 | 0.4307±0.0176^**^ | 0.1360±0.0034 | 0.4060±0.0199^*^ |
| D2 | 0.4020±0.0468^**^ | 0.6383±0.0152 | 0.5183±0.0245^**^ | 0.1487±0.0384 | 0.4020±0.0253^**^ |
| D3 | 0.7027±0.0140^**^ | 0.8503±0.0787 | 0.8300±0.0242^**^ | 0.3270±0.0496 | 0.7120±0.0285^**^ |
| D4 | 0.7350±0.0205^**^ | 0.8013±0.0184 | 0.8080±0.0220^**^ | 0.4100±0.0121 | 0.8047±0.0149 |
| D5 | 0.8057±0.0527^**^ | 0.9280±0.0602 | 1.0020±0.0824^**^ | 0.4387±0.0288 | 0.8717±0.0140^*^ |

NC①: miR-27a mimic NC, siR-sFRP1 NC; NC②: miR-27a inhibitor NC; **p* ≤0.05*；**p* ≤0.01. hFOBs were cultured in non-differentiation medium at 33.4 ℃ for up to 5 days.
