# Supplementary material for: MiR-27a Targets sFRP1 in hFOB Cells to Regulate Proliferation, Apoptosis and Differentiation
Source: PLoS One. 2014 Mar 13;9(3):e91354. doi: 10.1371/journal.pone.0091354 (PMC3953332; doi:10.1371/journal.pone.0091354)
Supplement: Table S5 — The inhibitory effect of sFRP1 knockdown on hFOBs proliferation can be reversed by the knockdown of miR-27a. (non-differentiation in vitro) (OD450, Mean ± SD). (DOC) [file pone.0091354.s006.doc]

**Table S5. The inhibitory effect of sFRP1 knockdown on hFOBs proliferation can be reversed by the knockdown of miR-27a.** (non-differentiation *in vitro*) (OD450, Mean ± SD).

|  | siR-sFRP1 + miR-27a inhibitor | NC^③^ |
| --- | --- | --- |
| D1 | 0.3740 ± 0.0027^*^ | 0.3223 ± 0.0045 |
| D2 | 0.4163 ± 0.0150^**^ | 0.3590 ± 0.0217 |
| D3 | 0.6583 ± 0.0185^*^ | 0.5573 ± 0.0181 |
| D4 | 0.7480 ± 0.0156^*^ | 0.6033 ± 0.0680 |
| D5 | 0.7533 ± 0.0436^*^ | 0.6643 ± 0.0349 |

NC③: siR-sFRP1 + miR-27a inhibitor NC；**p* ≤0.05；***p* ≤0.01*.* hFOBs were cultured in non-differentiation medium at 33.4 ℃ for up to 5 days.
