# Supplementary material for: MiR-27a Targets sFRP1 in hFOB Cells to Regulate Proliferation, Apoptosis and Differentiation
Source: PLoS One. 2014 Mar 13;9(3):e91354. doi: 10.1371/journal.pone.0091354 (PMC3953332; doi:10.1371/journal.pone.0091354)
Supplement: Table S6 — The influence of miR-27a/sFRP1 on hFOB proliferation. (osteogenic differentiation in vitro). (OD450, Mean ± SD). (DOC) [file pone.0091354.s007.doc]

**Table S6. The influence of miR-27a/sFRP1 on hFOB proliferation. (osteogenic differentiation *in vitro).*** (OD450, Mean ± SD)

|  | miR-27a mimic | NC^①^ | miR-27a inhibitor | NC^②^ | siR-sFRP1 |
| --- | --- | --- | --- | --- | --- |
| D1 | 0.2520±0.0020^**^ | 0.4950±0.0590 | 0.3600±0.0110^**^ | 0.2530±0.0100 | 0.3210± 0.0110^**^ |
| D2 | 0.2610±0.0108^**^ | 0.5000±0.0072 | 0.3230±0.0144^**^ | 0.2327±0.0073 | 0.2913±0.02120^**^ |
| D3 | 0.2860±0.0061^**^ | 0.9180±0.0825 | 0.5200±0.0295^**^ | 0.2893±0.0135 | 0.3973±0.0121^**^ |
| D4 | 0.2980±0.0090^**^ | 0.7613±0.0100 | 0.5653±0.0348^**^ | 0.3187±0.0107 | 0.4417±0.0188^**^ |
| D5 | 0.3040±0.0642^**^ | 0.6163±0.0316 | 0.3773±0.0170^**^ | 0.2533±0.0128 | 0.3470±0.0242^**^ |

NC①: miR-27a mimic NC, siR-sFRP1 NC; NC②: miR-27a inhibitor NC; inhibitor NC；**p* ≤0.05*；**p* ≤0.01 **.** hFOBs were cultured in osteogenic medium at 39.4 ℃ for up to 5 days.
