# Supplementary material for: MiR-27a Targets sFRP1 in hFOB Cells to Regulate Proliferation, Apoptosis and Differentiation
Source: PLoS One. 2014 Mar 13;9(3):e91354. doi: 10.1371/journal.pone.0091354 (PMC3953332; doi:10.1371/journal.pone.0091354)
Supplement: Table S7 — The inhibitory effect of sFRP1 knockdown on hFOB proliferation can be reversed by the knockdown of miR-27a. (osteogenic differentiation in vitro) (OD450, Mean ± SD). (DOC) [file pone.0091354.s008.doc]

**Table S7. The inhibitory effect of sFRP1 knockdown on hFOB proliferation can be reversed by the knockdown of miR-27a.** (osteogenic differentiation *in vitro*) (OD450, Mean ± SD)

|  | siR-sFRP1+miR-27a inhibitor | NC^③^ |
| --- | --- | --- |
| D1 | 0.3460 ± 0.0140 ^**^ | 0.2840 ± 0.0080 |
| D2 | 0.3297 ± 0.0060 ^**^ | 0.2653 ± 0.0047 |
| D3 | 0.4343 ± 0.0317 ^**^ | 0.3003 ± 0.0075 |
| D4 | 0.4383 ± 0.0327 ^**^ | 0.3357 ± 0.0077 |
| D5 | 0.3257 ± 0.0083 ^**^ | 0.2897 ± 0.0136 |

NC③: siR-sFRP1 + miR-27a inhibitor NC；**p* ≤0.05*；**p* ≤0.01*.* hFOBs were cultured in osteogenic medium at 39.4 ℃ for up to 5 days.
